# Isotope tracing reveals bacterial catabolism of host-derived glutathione during *Helicobacter pylori* infection

**DOI:** 10.1371/journal.ppat.1011526

**Published:** 2023-07-26

**Authors:** Maia J. Baskerville, Yekaterina Kovalyova, Raquel Mejías-Luque, Markus Gerhard, Stavroula K. Hatzios

**Affiliations:** 1 Department of Microbial Pathogenesis, Yale University School of Medicine, New Haven, Connecticut, United States of America; 2 Department of Molecular, Cellular, and Developmental Biology, Yale University, New Haven, Connecticut, United States of America; 3 Microbial Sciences Institute, Yale University, West Haven, Connecticut, United States of America; 4 Department of Chemistry, Yale University, New Haven, Connecticut, United States of America; 5 Institute of Medical Microbiology, Immunology and Hygiene, Technical University of Munich, School of Medicine, Munich, Germany; 6 German Centre for Infection Research, Munich, Germany; University of Toronto, CANADA

## Abstract

Mammalian cells synthesize the antioxidant glutathione (GSH) to shield cellular biomolecules from oxidative damage. Certain bacteria, including the gastric pathogen *Helicobacter pylori*, can perturb host GSH homeostasis. *H*. *pylori* infection significantly decreases GSH levels in host tissues, which has been attributed to the accumulation of reactive oxygen species in infected cells. However, the precise mechanism of *H*. *pylori-*induced GSH depletion remains unknown, and tools for studying this process during infection are limited. We developed an isotope-tracing approach to quantitatively monitor host-derived GSH in *H*. *pylori-*infected cells by mass spectrometry. Using this method, we determined that *H*. *pylori* catabolizes reduced GSH from gastric cells using γ-glutamyl transpeptidase (gGT), an enzyme that hydrolyzes GSH to glutamate and cysteinylglycine (Cys-Gly). gGT is an established virulence factor with immunomodulatory properties that is required for *H*. *pylori* colonization *in vivo*. We found that *H*. *pylori* internalizes Cys-Gly in a gGT-dependent manner and that Cys-Gly production during *H*. *pylori* infection is coupled to the depletion of intracellular GSH from infected cells. Consistent with bacterial catabolism of host GSH, levels of oxidized GSH did not increase during *H*. *pylori* infection, and exogenous antioxidants were unable to restore the GSH content of infected cells. Altogether, our results indicate that *H*. *pylori-*induced GSH depletion proceeds via an oxidation-independent mechanism driven by the bacterial enzyme gGT, which fortifies bacterial acquisition of nutrients from the host. Additionally, our work establishes a method for tracking the metabolic fate of host-derived GSH during infection.

## Author summary

Oxidative stress is a common outcome of microbial infections that can cause extensive damage to cellular biomolecules. Host cells mitigate oxidative damage by producing the antioxidant glutathione. The gastric pathogen *Helicobacter pylori* decreases glutathione levels in gastric cells through an unknown mechanism, which makes host tissues more vulnerable to harmful oxidants. To monitor glutathione dynamics during infection, we developed an isotope-labeling strategy that can track host-derived glutathione in *H*. *pylori-*infected cells. We found that *H*. *pylori* degrades host glutathione using the enzyme γ-glutamyl transpeptidase, facilitating bacterial uptake of the glutathione-derived dipeptide cysteinylglycine. Our findings demonstrate that *H*. *pylori* exploits a protective metabolite from the host for nutritional gain during infection.

## Introduction

Oxidative stress results from an imbalance between the production and detoxification of reactive oxygen species (ROS) in a biological system. ROS production is a hallmark of microbial infections, with epithelial barriers, phagocytic immune cells, and pathogen-associated virulence factors all contributing to oxidative stress at the host–microbe interface. ROS accumulation in infected tissues can induce the oxidation of important cellular components including proteins, lipids, and DNA [1,2]. Host cells shield these biomolecules from oxidative damage in part by synthesizing the antioxidant glutathione (GSH) [2]. As the most abundant molecular thiol in eukaryotes, GSH is essential for the detoxification of ROS, the reduction of protein thiols, and the maintenance of intracellular redox homeostasis [3]. Consequently, GSH deficiency has been associated with severe pathophysiological outcomes including neurodegenerative diseases [4–6] and metabolic disorders [7–9].

Microbial utilization of host GSH during infection can also contribute to disease. The facultative intracellular pathogen *Francisella tularensis* metabolizes host GSH to support its replication within mammalian cells [10]. *Listeria monocytogenes* uses host GSH as an environmental cue to regulate virulence gene expression [11,12]. Several extracellular pathogens, including *Streptococcus mutans* [13] and *Campylobacter jejuni* [14,15], consume exogenous GSH for nutritional purposes. While genetic approaches have been valuable for identifying bacterial genes required for GSH import and metabolism, tools for directly assessing bacterial utilization of host GSH remain limited. To date, most studies have relied on metabolic labeling of bacterial cultures with radiolabeled GSH to measure GSH uptake [16]. The ability to directly monitor bacterial metabolism of host-derived GSH in the context of infection would provide new mechanistic insights into microbial utilization of this important cellular thiol.

The gastric pathogen *Helicobacter pylori*, a major cause of peptic ulcers and the leading risk factor for gastric cancer [17–19], offers a powerful model system for studying GSH dynamics at the host–microbe interface. *H*. *pylori* infection significantly decreases GSH levels in human gastric cells and tissues [20–25], which has been attributed to the persistent generation of ROS during infection [20,23,25]. *H*. *pylori* induces ROS production by recruiting ROS-generating immune cells to infected tissues, stimulating the activity of host oxidases, and secreting virulence factors that increase ROS levels within infected cells [20,26,27]. Oxidative stress plays a significant role in the development of *H*. *pylori-*induced gastric pathology and cancer; indeed, the genetic disruption of host oxidases [28], or the oral administration of antioxidants [29], significantly reduces gastric inflammation [29], colonization [30], and/or tumor formation [31] in rodent models of infection. While the oxidizing conditions that accompany *H*. *pylori* infection have been hypothesized to lower host GSH levels by converting GSH to its oxidized form [20], the precise mechanism underlying host GSH depletion remains unresolved.

In this study, we combined isotope tracing with reactivity-guided metabolomics to quantify the abundance and metabolism of host-derived GSH during *H*. *pylori* infection. We found that the *H*. *pylori* enzyme γ-glutamyl transpeptidase (gGT), which hydrolyzes GSH to glutamate and cysteinylglycine (Cys-Gly) [16,32], is required for the depletion of intracellular GSH from *H*. *pylori-*infected cells. Our results indicate that GSH depletion is driven by bacterial catabolism, and not by oxidation, of this abundant host thiol during *H*. *pylori* infection.

## Results

### *H*. *pylori* depletes GSH from gastric cells

**Fig 1:**
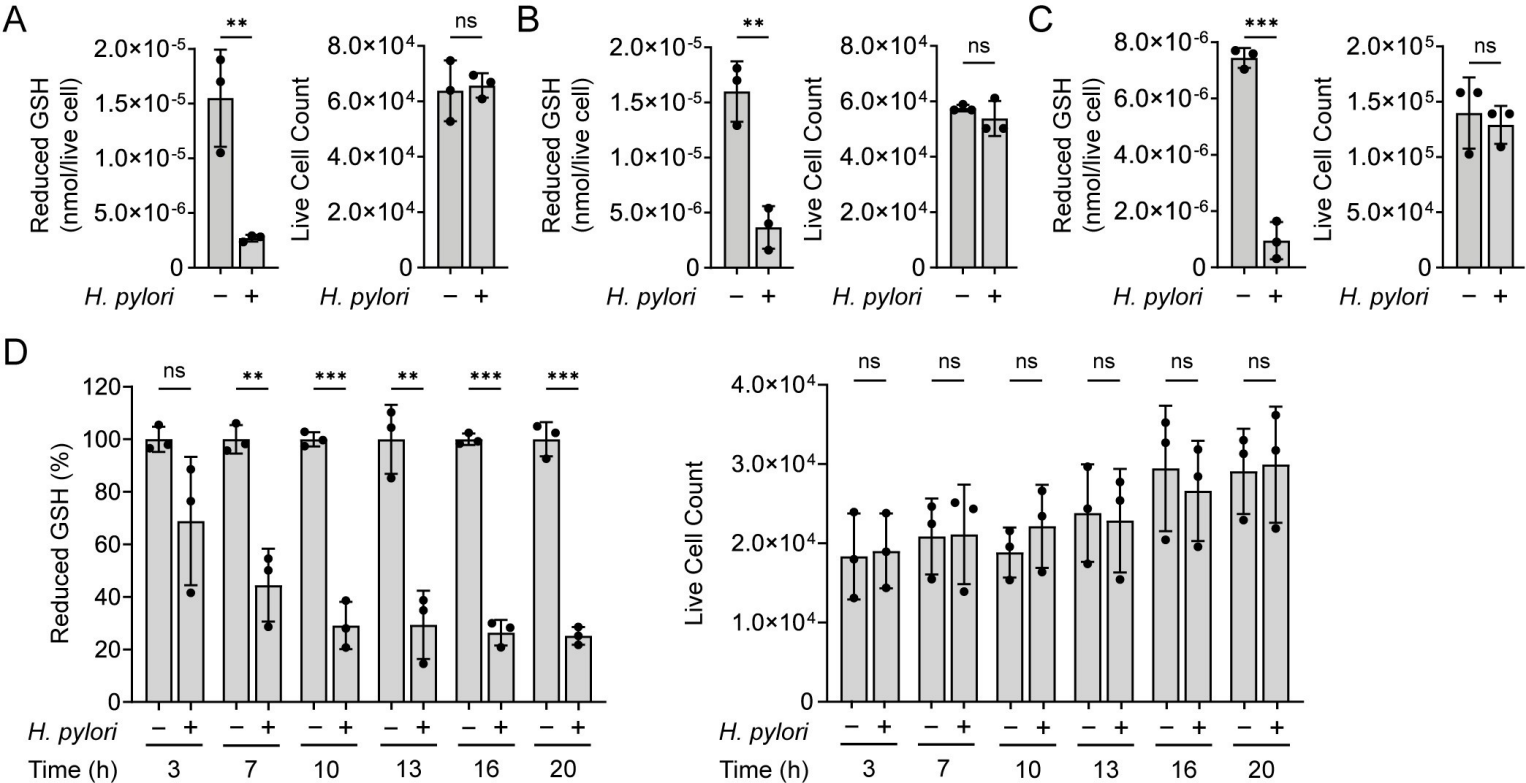
*H*. *pylori* infection depletes reduced GSH from gastric cells. Intracellular reduced GSH levels in *H*. *pylori*-infected (*H*. *pylori* G27, MOI 50, 10 h) and uninfected (A) AGS, (B) MKN28, and (C) NCI-N87 gastric cells (left), normalized by the total number of live cells per condition (right). (D) Intracellular reduced GSH levels in *H*. *pylori*-infected (*H*. *pylori* G27, MOI 50) and uninfected AGS cells at 3, 7, 10, 13, 16, and 20 h post infection (left), normalized by the total number of live cells per condition (right). GSH data are expressed as a percentage of the average reduced GSH concentration in the corresponding uninfected control. Data represent three independent experiments. Each circle represents an independent experiment. Error bars represent means ± SD. **P < 0.01; ***P < 0.001; ns, not significant. Two-tailed unpaired t-tests were used for (A-C) and multiple unpaired t-tests were used for (D).

We measured the concentration of reduced GSH in three gastric cancer cell lines (AGS, MKN28, and NCI-N87) infected with *H*. *pylori* strain G27. Consistent with prior studies [20–25], we observed a significant decrease in the intracellular GSH concentration of *H*. *pylori-*infected cells relative to uninfected controls (Fig 1A–1C). Both infected and uninfected cells were equally viable under the tested conditions (multiplicity of infection (MOI) 50 for 10 h), demonstrating that GSH depletion during *H*. *pylori* infection was not due to an overall decrease in gastric cell viability. GSH levels decreased over time in *H*. *pylori-*infected AGS cells, with significantly lower concentrations evident as early as 7 h post infection (Fig 1D). These findings indicate that *H*. *pylori* infection induces a progressive decline in host GSH levels, independent of host cell death.

### *H*. *pylori* does not increase GSH oxidation in gastric cells

**Fig 2:**
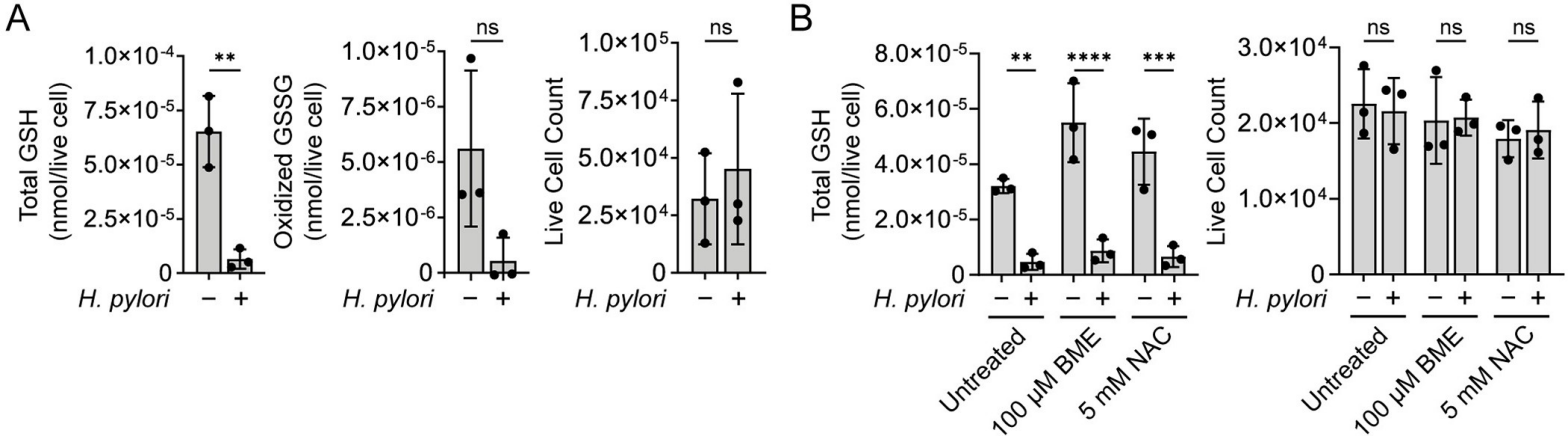
*H*. *pylori* infection does not increase GSH oxidation in gastric cells. (A) Levels of total GSH (left) or oxidized GSSG (middle) in *H*. *pylori*-infected (*H*. *pylori* G27, MOI 50, 10 h) and uninfected AGS cells, normalized by the total number of live AGS cells in each condition (right). GSH and GSSG measurements were performed on the same population of cells. (B) Levels of total GSH in *H*. *pylori*-infected (*H*. *pylori* G27, MOI 50, 10 h) and uninfected AGS cells pre-incubated with 2-mercaptoethanol (BME), *N*-acetylcysteine (NAC), or medium alone (untreated) for 1 h (left), normalized by the total number of live AGS cells in each condition (right). AGS cells were also incubated with BME, NAC, or medium alone for the duration of infection. Data represent three independent experiments. Each circle represents an independent experiment. Error bars represent means ± SD. **P < 0.01; ***P < 0.001; ****P < 0.0001; ns, not significant. A two-tailed unpaired t-test was used for (A), and a two-way ANOVA with Šídák’s multiple comparisons test was used for (B).

GSH depletion during *H*. *pylori* infection has previously been attributed to the accumulation of ROS within infected cells [20,23,25]. *H*. *pylori* infection is known to stimulate ROS production [25,33–35], which could in principle deplete intracellular pools of reduced GSH by generating the oxidized disulfide dimer GSSG. Indeed, *H*. *pylori-*infected AGS cells exhibit substantially higher levels of intracellular ROS than uninfected controls [25,33,34]. To determine whether oxidation is the primary cause of GSH depletion during *H*. *pylori* infection, we quantified total GSH (reduced GSH plus oxidized GSSG) levels in *H*. *pylori-*infected AGS cells. Similar to reduced GSH (Fig 1A–1C), we observed a significant decrease in total GSH following *H*. *pylori* infection (Fig 2A). Notably, GSSG levels did not increase in *H*. *pylori-*infected cells, suggesting that the observed depletion of total GSH was not due to GSH oxidation and instead driven by a specific decrease in reduced GSH. To validate our findings, we measured the total GSH concentration of *H*. *pylori-*infected cells treated with the potent reducing agents 2-mercaptoethanol or *N*-acetylcysteine. Treatment with 2-mercaptoethanol increased the overall GSH concentration of uninfected cells (Fig 2B, compare bars 1 and 3; S1 Fig), as previously described [36]; however, neither reducing agent inhibited the loss of GSH induced by *H*. *pylori* infection (Fig 2B). Taken together, these results support that *H*. *pylori* infection depletes host GSH via an oxidation-independent mechanism.

### *H*. *pylori* gGT is required for host GSH depletion

**Fig 3:**
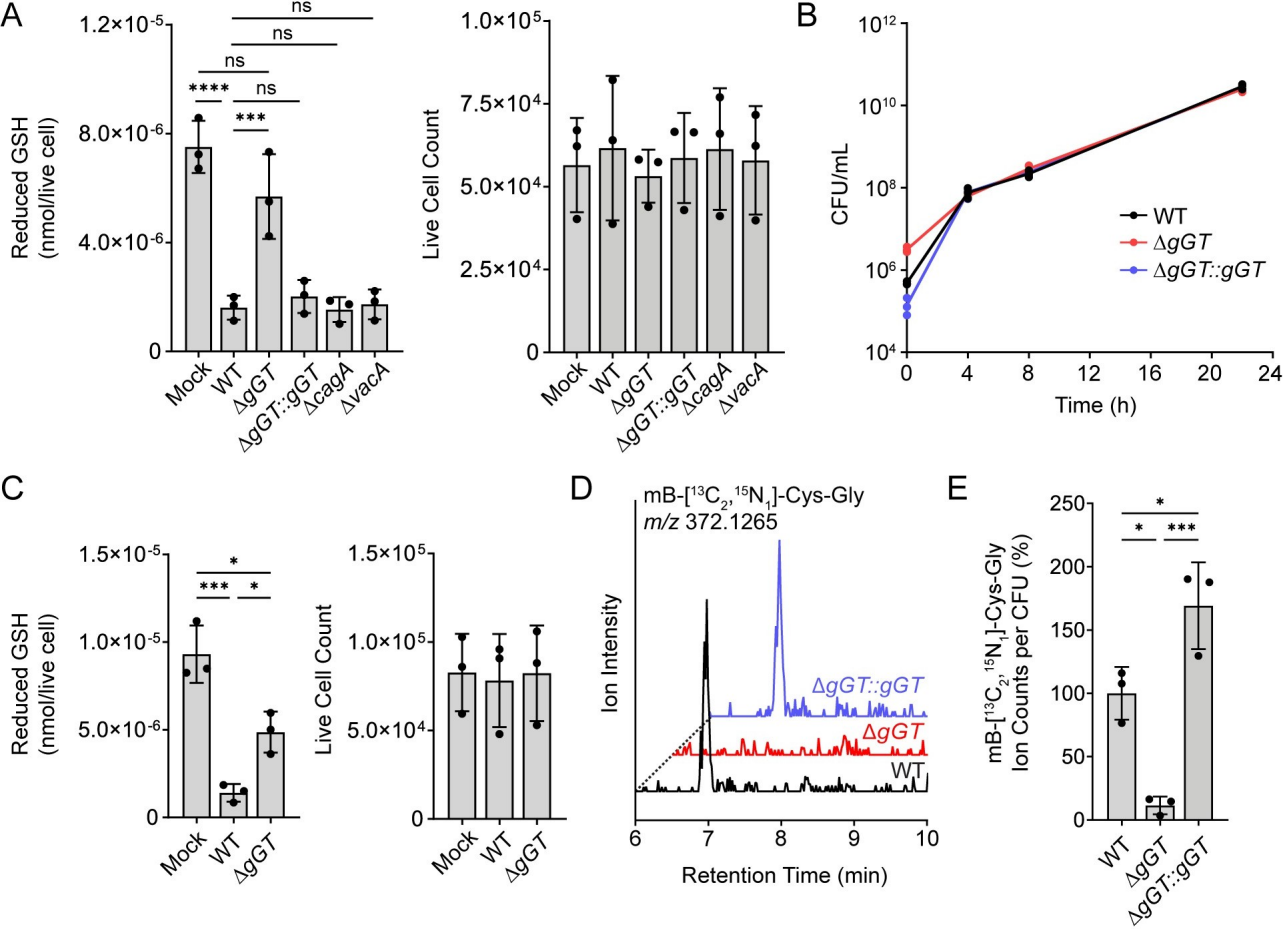
*H*. *pylori* gGT is required for host GSH depletion and bacterial uptake of Cys-Gly. *(*A) Reduced GSH levels in AGS cells infected with WT, Δ*gGT*, Δ*gGT∷gGT*, Δ*cagA*, Δ*vacA H*. *pylori* G27 (MOI 35) or medium alone (mock) for 16 h (left), normalized by the total number of live cells per condition (right). (B) CFU from independent cultures of WT, Δ*gGT*, and Δ*gGT∷gGT H*. *pylori* G27 were enumerated at the indicated time points. (C) Reduced GSH levels in AGS cells infected with WT, Δ*gGT H*. *pylori* PMSS1 (MOI 35) or medium alone (mock) for 16 h (left), normalized by the total number of live cells per condition (right). (D) WT, Δ*gGT*, or Δ*gGT∷gGT H*. *pylori* G27 were cultured in medium supplemented with heavy GSH ([^13^C_2_, ^15^N_1_]-GSH) for 8 h. Extracted ion chromatogram (EIC) spectra (*m/z* 372.1265, corresponding to mB-[^13^C_2_, ^15^N_1_]-Cys-Gly) of *H*. *pylori* cell extracts treated with mBBr. (E) Ion counts of mB-[^13^C_2_, ^15^N_1_]-Cys-Gly normalized by CFU and expressed as a percentage of the average ion counts per CFU for the WT strain. Data in (A), (C), (D), and (E) represent three independent experiments. Data in (B) represent three replicates of a single experiment. Each circle represents a technical replicate. Growth curve analyses were performed twice with consistent results. Each circle in (A), (C), and (E) represents an independent experiment. Error bars represent means ± SD. *P < 0.05; ***P < 0.001; ****P < 0.0001; ns, not significant. A one-way ANOVA with Šídák’s multiple comparisons test was used for (A) and (C), and a one-way ANOVA with Dunnett’s multiple comparisons test was used for (E).

As an extracellular gastric pathogen, *H*. *pylori* has access to dietary nutrients in the stomach and relies on the uptake of host amino acids for survival [37–40]. The import of extracellular glutamine is facilitated by its deamidation to glutamate, a process catalyzed by the periplasmic enzyme gGT [41]. gGT also generates glutamate through the hydrolysis of γ-glutamyl compounds including GSH, which is converted to glutamate and Cys-Gly [16,32]. Although gGT is known to hydrolyze extracellular GSH, it is unknown whether this enzyme contributes to GSH depletion within gastric cells infected by *H*. *pylori*. To test this, we compared the amount of reduced GSH in AGS cells infected with wild-type (WT) *H*. *pylori* G27, an isogenic *gGT* deletion mutant (Δ*gGT*), or a genetically complemented mutant strain (Δ*gGT*::*gGT*). Consistent with our prior findings (Fig 1A–1C), we detected significantly lower levels of reduced GSH in cells infected with WT *H*. *pylori* relative to uninfected controls (Fig 3A; MOI 35 for 16 h). By contrast, cells infected with the Δ*gGT* mutant exhibited much higher GSH levels, comparable to those observed in uninfected cells. Genetic complementation restored *gGT* expression (S2 Fig) and GSH-depleting activity (Fig 3A) to the Δ*gGT* mutant. Notably, infection with *H*. *pylori* mutants lacking the secreted virulence factors cytotoxin-associated gene A (Δ*cagA*) or vacuolating cytotoxin A (Δ*vacA*), which have previously been shown to stimulate ROS production in infected cells [25,35,42], diminished host GSH by the same extent as WT *H*. *pylori*. These findings underscore that GSH depletion is not driven by oxidation under the tested conditions (Fig 2). In addition, because the WT, Δ*gGT*, and Δ*gGT*::*gGT* strains exhibited uniform growth kinetics when cultured individually (Fig 3B), and all strains were similarly viable when co-cultured with AGS cells (S3 Fig), the observed changes in host GSH levels were not due to differences in the viability of the tested strains. We detected similar changes in the abundance of host GSH using AGS cells infected with a second WT strain of *H*. *pylori*, PMSS1, and an isogenic *gGT* deletion mutant (Fig 3C). Collectively, these data demonstrate that gGT contributes to the depletion of intracellular GSH from gastric cells during *H*. *pylori* infection.

### gGT facilitates *H*. *pylori* uptake of Cys-Gly

Although gGT-mediated hydrolysis of extracellular GSH is known to facilitate *H*. *pylori* uptake of glutamate [16], it is unknown whether *H*. *pylori* also utilizes the other product of GSH degradation, Cys-Gly, as a nutrient source. Notably, GSH degradation is a mechanism employed by the intracellular pathogen *F*. *tularensis* to scavenge Cys-Gly from the host cytosol [43]. Similarly, we hypothesized that *H*. *pylori* gGT may mediate bacterial acquisition of Cys-Gly from extracellular GSH. To test this, we cultured WT, Δ*gGT*, and Δ*gGT*::*gGT H*. *pylori* in rich medium supplemented with isotopically labeled [^13^C_2_, ^15^N_1_]-GSH and quantified the relative abundance of [^13^C_2_, ^15^N_1_]-Cys-Gly in bacterial extracts by liquid chromatography-mass spectrometry (LC-MS). All three strains grew equally well following supplementation with isotopically light GSH or [^13^C_2_, ^15^N_1_]-GSH (S4 and S5 Figs), suggesting that neither cysteine, glycine, nor glutamate were limiting under the tested conditions. Bacterial cell extracts were treated with the thiol-alkylating agent monobromobimane (mBBr) to facilitate the detection of Cys-Gly and other thiol-containing metabolites. The bimane derivative of [^13^C_2_, ^15^N_1_]-Cys-Gly was readily detected in WT and Δ*gGT*::*gGT H*. *pylori* extracts, but was present at much lower levels in extracts of the Δ*gGT* mutant (Fig 3D and 3E). These results demonstrate that *H*. *pylori* gGT facilitates bacterial acquisition of Cys-Gly from extracellular GSH and suggest that the depletion of host GSH by *H*. *pylori* may enable bacterial consumption of Cys-Gly during infection.

### *H*. *pylori* metabolizes GSH produced by gastric cells

**Fig 4:**
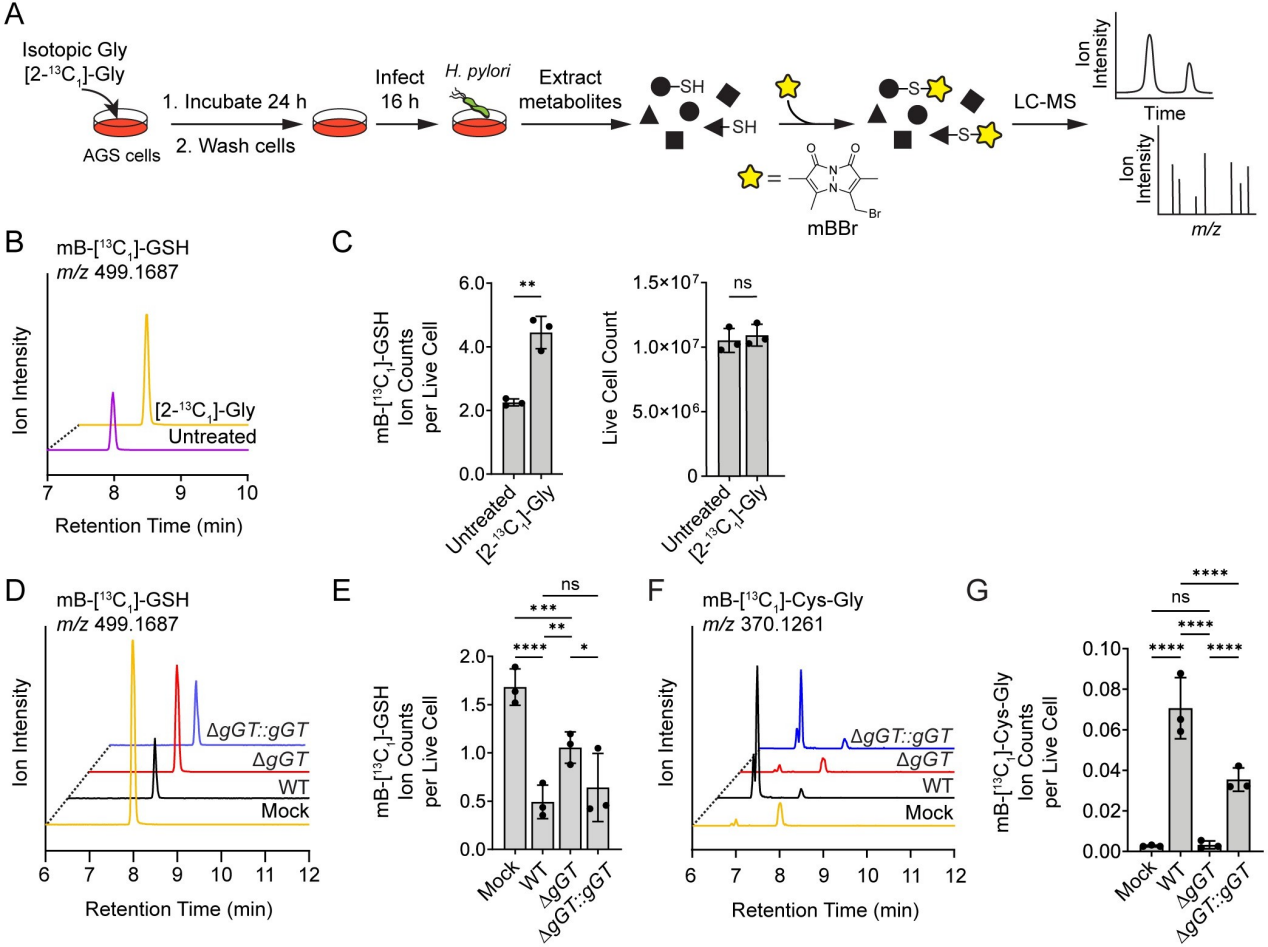
*H*. *pylori* catabolizes host-derived GSH. (A) Workflow for the detection of isotopically labeled GSH and Cys-Gly in AGS cells infected with *H*. *pylori*. AGS cells were incubated with [2-^13^C_1_]-Gly for 24 h, washed, and infected with WT, Δ*gGT*, or Δ*gGT∷gGT H*. *pylori* G27 for 16 h (MOI 35). Cell extracts were treated with mBBr and analyzed by LC-MS. (B) AGS cells were incubated with [2-^13^C_1_]-Gly or medium alone (untreated) for 24 h. EIC spectra (*m/z* 499.1687, corresponding to mB-[^13^C_1_]-GSH) of AGS cell extracts treated with mBBr. (C) Ion counts of mB-[^13^C_1_]-GSH for (*B*) (left), normalized by the total number of live cells per condition (right). (D) AGS cells were incubated with [2-^13^C_1_]-Gly for 24 h and infected with WT, Δ*gGT*, or Δ*gGT∷gGT H*. *pylori* G27 (MOI 35) or medium alone (mock) for 16 h. EIC spectra (*m/z* 499.1687, corresponding to mB-[^13^C_1_]-GSH) of AGS cell extracts treated with mBBr. (E) Ion counts of mB-[^13^C_1_]-GSH for (D), normalized by the total number of live cells per condition. (F) AGS cells were incubated with [2-^13^C_1_]-Gly for 24 h and infected with WT, Δ*gGT*, or Δ*gGT∷gGT H*. *pylori* G27 (MOI 35) or medium alone (mock) for 16 h. EIC spectra (*m/z* 370.1261, corresponding to mB-[^13^C_1_]-Cys-Gly; retention time 6.9 min) of AGS cell extracts treated with mBBr. (G) Ion counts of mB-[^13^C_1_]-Cys-Gly for (F), normalized by the total number of live cells per condition. LC-MS analyses in (D-G) were performed using the same population of cells. Data in (B-G) represent three independent experiments. Each circle in (C), (E), and (G) represent*s* an independent experiment. Error bars represent means ± SD. *P < 0.05; **P < 0.01; ***P < 0.001; ****P < 0.0001; ns, not significant. A two-tailed unpaired t-test was used for (C), and a two-way ANOVA with Šídák’s multiple comparisons test was used for (E) and (G).

We developed an isotope-tracing assay to determine whether host GSH is hydrolyzed by gGT during *H*. *pylori* infection (Fig 4A). Previous studies have shown that cells supplemented with isotopically heavy glycine can use native biosynthetic pathways (S6 Fig) to generate heavy GSH [44–46]. By applying LC-MS to quantify the relative abundance of heavy GSH and heavy Cys-Gly in *H*. *pylori-*infected cells, we reasoned we could directly measure bacterial metabolism of host GSH during infection.

To generate heavy GSH, AGS cells were cultured in medium supplemented with increasing concentrations of heavy glycine ([2-^13^C_1_]-Gly; 0, 10, 25, or 50 μg/mL) for 24 h. The relative abundance of mB-[^13^C_1_]-GSH in AGS cell extracts was quantified by mBBr labeling and LC-MS analysis (S7A and S7B Fig). Cells supplemented with 50 μg/mL [2-^13^C_1_]-Gly exhibited a significant increase in heavy GSH relative to untreated controls, with no apparent decrease in cell viability (Figs 4B, 4C, S7A and S7B). The bimane adduct of heavy GSH exhibited the same fragmentation pattern as an mBBr-treated standard of isotopically light GSH when analyzed by liquid chromatography-tandem mass spectrometry (LC-MS/MS), with the expected mass shift of +1 amu (S7C Fig). Notably, due to the natural isotopic distribution of light GSH (S7D and S7E Fig), mB-[^13^C_1_]-GSH (*m/z* 499.1687) was also detected in untreated cells (Fig 4B and 4C). However, the ~2-fold increase in signal afforded by [2-^13^C_1_]-Gly supplementation was sufficient to quantify differences in heavy GSH abundance in subsequent assays.

To assess bacterial degradation of heavy GSH, we pretreated AGS cells with 50 μg/mL [2-^13^C_1_]-Gly for 24 h, then washed the cells to remove exogenous [2-^13^C_1_]-Gly prior to infection with WT, Δ*gGT*, or Δ*gGT*::*gGT H*. *pylori* G27 (MOI 35 for 16 h) (Fig 4A). The relative abundance of heavy GSH and heavy Cys-Gly in *H*. *pylori-*infected cell extracts was quantified by mBBr labeling and LC-MS analysis. Consistent with our findings using a commercial assay for GSH quantitation (Fig 3A), we detected considerably lower levels of mB-[^13^C_1_]-GSH in cells infected with WT or Δ*gGT*::*gGT H*. *pylori* compared to cells infected with the mutant strain (Figs 4D, 4E and S8). Accordingly, we hypothesized that [^13^C_1_]-Cys-Gly levels would be greatest in extracts from cells infected with WT or Δ*gGT*::*gGT H*. *pylori*, reflecting gGT-dependent hydrolysis of [^13^C_1_]-GSH. Indeed, significant levels of [^13^C_1_]-Cys-Gly were only detected in the presence of gGT-encoding *H*. *pylori* strains (Fig 4F and 4G). Extracts from cells infected with Δ*gGT H*. *pylori* or uninfected controls contained almost no [^13^C_1_]-Cys-Gly, suggesting that the production of this metabolite stems from the GSH-degrading activity of adherent bacteria. Notably, we were unable to detect [^13^C_1_]-Cys-Gly in nonadherent bacteria harvested from the cell culture medium under these conditions (S9 Fig). Altogether, our results demonstrate that gGT degrades host GSH during *H*. *pylori* infection and that this process contributes to the depletion of GSH within infected cells.

## Discussion

In this study, we found that *H*. *pylori* catabolizes host GSH, resulting in the depletion of this protective thiol from *H*. *pylori-*infected gastric cells. Although oxidative stress has previously been cited as the presumptive cause of GSH depletion during *H*. *pylori* infection [20,23], our findings support a model wherein *H*. *pylori* uses the periplasmic enzyme gGT, an established virulence factor, to degrade and acquire nutrients from host GSH. Extensive work by several groups over the past two decades has uncovered multiple immunomodulatory roles for gGT during *H*. *pylori* infection, including the inhibition of T-cell proliferation [47] and induction of dendritic cell tolerance [48]. However, to date, no direct link between this enzyme and *H*. *pylori* consumption of host GSH has been established.

Our data position gGT at the helm of a nutrient acquisition system that supports bacterial survival in the stomach while corrupting the ability of host cells to maintain intracellular redox homeostasis. Given that GSH is typically maintained at millimolar levels within gastric tissues [49], this tripeptide constitutes a significant reservoir of cysteine and glutamate in the host environment. Indeed, exogenous peptides and amino acids serve as an important carbon source for *H*. *pylori* [37,38]. Extracellular glutamate is incorporated into the *H*. *pylori* tricarboxylic acid cycle [16], and we found that Cys-Gly, the other product of GSH hydrolysis [16,32], is also internalized by *H*. *pylori* (Fig 3E). Although GSH supplementation did not enhance the growth of gGT-expressing strains in broth cultures (S4 Fig), the rich medium required for *H*. *pylori* growth could mask nutritional advantages conferred by GSH metabolism *in vitro* [50]. Nevertheless, given that the expression of gGT is required for *H*. *pylori* colonization of murine gastric tissues [48,51], our findings imply that degradation of host GSH supports *H*. *pylori* metabolism and survival *in vivo*.

While our data establish a role for *H*. *pylori* gGT in depleting GSH from gastric cells, it remains unclear how *H*. *pylori* gains access to intracellular pools of host GSH. The detection of host-derived Cys-Gly in AGS cells infected with WT or Δ*gGT*::*gGT H*. *pylori*, but not in cells infected with the Δ*gGT* mutant (Fig 4G) or in WT bacteria harvested from conditioned co-culture medium (S9 Fig), suggests bacterial degradation of host GSH may be contact-dependent. Host cell lysis induced by *H*. *pylori* infection could make intracellular GSH available for bacterial consumption. Alternatively, *H*. *pylori* may actively translocate GSH from, or stimulate GSH efflux by, infected cells as a means of promoting GSH catabolism by adherent bacteria. Additional studies are needed to determine whether specific *H*. *pylori* or host transporters facilitate bacterial access to intracellular GSH.

Although gGT appears to be the principal driver of host GSH depletion during *H*. *pylori* infection, other bacterial and/or host factors may augment this phenotype. Indeed, in certain experiments, we detected lower GSH levels in cells infected with the Δ*gGT* mutant than in uninfected controls (Figs 3C and 4E). Prior studies indicate that the expression of cation transport regulator 1 (CHAC1), a host enzyme that cleaves GSH into 5-oxoproline and Cys-Gly [52], is elevated in *H*. *pylori-*infected gastric cells under certain conditions [53,54]. However, there have been conflicting reports regarding the transcriptional regulation of *CHAC1* during infection [55], and our data suggest that hydrolysis of host GSH to Cys-Gly in *H*. *pylori-*infected cells is gGT-dependent (Fig 4G). Similarly, the secreted *H*. *pylori* virulence factor VacA has been reported to decrease levels of host GSH by increasing the accumulation of intracellular ROS [25], though our data do not support a role for VacA in GSH depletion under the conditions tested here (Fig 3A). Nevertheless, strain-specific factors and infection conditions may influence the mechanisms of GSH depletion that are active at the host–microbe interface.

Our work establishes the utility of isotope tracing and mass spectrometry in tracking GSH metabolism during infection. This approach permits the precise monitoring of host-synthesized GSH and its degradation product Cys-Gly in *H*. *pylori-*infected cells, thereby offering a powerful complement to conventional radiolabeling assays. While radiolabeled GSH has been extensively used to detect cellular uptake of GSH [56–58], radiolabeling approaches can only provide a nonspecific readout of GSH metabolism based on the intracellular accumulation and/or proteome-wide incorporation of a specified radionuclide. These methods are generally unable to resolve the exact metabolites resulting from degradation of a radiolabeled precursor. Furthermore, because it is difficult to assess the distribution of radioactivity in a complex system, radioisotope-based approaches have thus far been limited to studies of GSH uptake by bacteria cultured independently *in vitro* [10,16,43]. By contrast, isotope tracing coupled to LC-MS enables the targeted analysis of host-derived Cys-Gly in infected cells and provides a safer alternative to radiolabeling approaches. Given the growing list of pathogens that co-opt host GSH to fortify bacterial metabolism [10,13–15,59] or activate virulence mechanisms during infection [12,60–62], this approach should be broadly useful for future studies of GSH utilization by bacteria in the host environment.

In summary, our findings redefine *H*. *pylori-*induced depletion of host GSH as a microbe-driven metabolic process that facilitates bacterial acquisition of essential amino acids. GSH degradation depletes gastric cells of this protective thiol, sensitizing host tissues to oxidative damage. As such, GSH depletion by *H*. *pylori* not only dynamically shapes redox homeostasis at the host–microbe interface, but comprises a previously unappreciated mechanism of ‘nutritional virulence’ [63] that could potentially be targeted to mitigate *H*. *pylori-*associated pathology.

## Materials and methods

### *H*. *pylori* strains and culture conditions

A complete list of the strains, plasmids, and primers used in this study can be found in S1–S3 Tables. *H*. *pylori* was grown on Columbia blood agar (Difco) plates containing 5% (v/v) defibrinated horse blood (Hemostat Labs), 50 μg/mL cycloheximide (Sigma), 10 μg/mL vancomycin (Sigma), 5 μg/mL cefsulodin (Sigma), 2.5 U/mL polymyxin B (Sigma), 5 μg/mL trimethoprim (Sigma), 8 μg/mL amphotericin B (Fisher Scientific), 0.2% (w/v) β-cyclodextrin (Sigma), and 25 μg/mL chloramphenicol (Sigma) or 50 μg/mL kanamycin (Fisher Scientific) as needed at 37°C in a humidified 10% CO_2_ incubator for 2–3 days. Overnight cultures were prepared in vented T25 flasks (Falcon) using Brucella broth (Difco) containing 10% (v/v) heat-inactivated fetal bovine serum (FBS-HI; Gibco). Flasks were incubated for 16–18 h at 37°C with shaking at 100 rpm under microaerophilic conditions using a 2.5 L gas pack jar containing an Oxoid CampyGen sachet (Thermo Fisher).

### Mammalian cell lines and culture conditions

AGS cells (ATCC CRL-1739) were cultured in Dulbecco’s Modified Eagle Medium (DMEM; Gibco) supplemented with 10% (v/v) FBS-HI (Gibco) and 1% (v/v) Antibiotic-Antimycotic (ABAM; Gibco) at 37°C in a humidified 5% CO_2_ incubator. NCI-N87 cells (ATCC CRL-5822) were cultured in RPMI 1640 (Gibco) supplemented with 10% (v/v) FBS-HI and 1% (v/v) ABAM. MKN28 cells stably transfected with an empty pRc/CMV expression plasmid as described previously [64] were a gift from Dr. Osamu Nagano (Division of Gene Regulation, Institute of Advanced Medical Research, Keio University School of Medicine). MKN28 cells were grown in RPMI 1640 medium supplemented with 10% (v/v) FBS-HI (Biowest) and 1% (v/v) ABAM.

### Generation of *H*. *pylori* mutant strains

*H*. *pylori* mutant strains were generated via natural transformation of *H*. *pylori* G27 with linear PCR constructs containing the flanking regions of the targeted gene and an antibiotic resistance cassette as previously described [65,66]. To construct *H*. *pylori* G27 Δ*vacA*, ~600-base pair (bp) DNA sequences flanking *vacA* (GenBank HPG27_840) were PCR-amplified from *H*. *pylori* G27 genomic DNA (gDNA) using the primers MB-14 and MB-15 (upstream flank) and MB-16 and MB-17 (downstream flank, including 14 bp of the 3’ end of the *vacA* open reading frame (ORF)). The *Campylobacter coli* chloramphenicol resistance cassette (*cat*; GenBank M35190.1) was amplified from *H*. *pylori* G27MA Δ*cagA* DNA (gift of Manuel Amieva, Stanford University School of Medicine) using the primers MB-1 and MB-2. The resulting PCR products were gel-purified using the QIAQuick Gel Purification Kit (QIAGEN) and assembled into a single linear construct via splicing by overlap extension PCR using primers MB-14 and MB-17. The final PCR product was gel-purified using the QIAQuick Gel Purification Kit (QIAGEN) prior to transformation of *H*. *pylori* G27.

To construct *H*. *pylori* G27 Δ*cagA*, a linear deletion construct consisting of the *cat* resistance cassette flanked by the ~500-bp DNA sequences immediately upstream and downstream of *cagA* (GenBank HPG27_507) was amplified from *H*. *pylori* G27MA Δ*cagA* gDNA using the primers MB-71 and MB-74. The downstream flank of *cagA* included 43 bp of the 3’ end of the *cagA* ORF. The resulting PCR product was purified using the QIAQuick PCR Purification kit (QIAGEN) prior to transformation of *H*. *pylori* G27.

The *H*. *pylori* G27 Δ*gGT*::*gGT* complemented strain was generated via natural transformation of *H*. *pylori* G27 Δ*gGT* [47] with a linear PCR construct containing the ~500-bp DNA sequence immediately upstream of *gGT* (GenBank HPG27_1063) followed by the complete *gGT* coding region, the *cat* cassette, and the ~500-bp sequence immediately downstream of *gGT*. This construct was amplified from plasmid pUC57_*gGT*::*gGT-cat*, which was custom synthesized by GenScript, and purified using the QIAQuick Gel Purification Kit (QIAGEN) prior to transformation of *H*. *pylori* G27 *ΔgGT*. All mutant and complemented strains were confirmed by locus-specific PCR reactions and DNA sequencing.

To verify the loss of *gGT* expression by *H*. *pylori* G27 Δ*gGT*, total RNA was extracted from log-phase cultures (OD_600_ ~ 0.4) of WT, Δ*gGT*, and Δ*gGT∷gGT H*. *pylori* G27 using the Direct-zol RNA Miniprep Plus kit (Zymo Research) for reverse transcription-quantitative PCR (RT-qPCR) analysis. *gGT* mRNA was amplified using primers MJB-21 and MJB-22 and the KAPA SYBR FAST One-Step qRT-PCR kit (Kapa Biosystems) according to the manufacturer’s instructions. The *H*. *pylori* housekeeping gene *ppk* (polyphosphate kinase, GenBank HP_1010; primers ABS-75 and ABS-76; [67]) was used to normalize mRNA expression. Samples were analyzed using a CFX96 Real-Time PCR Detection System (Bio-Rad). Relative changes in *gGT* expression were determined using the ΔΔCt method [68].

### *H*. *pylori* infection of gastric cells

Gastric cancer cell lines were seeded at a density of 2 x 10^5^ cells in the appropriate medium (DMEM or RPMI 1640 supplemented with 10% (v/v) FBS-HI) 20–24 h prior to infection in either 24-well flat-bottom tissue culture-treated plates (Corning; luminescence-based GSH assay) or 100-mm tissue culture-treated dishes (Corning; LC-MS analyses). *H*. *pylori* overnight cultures were either diluted to OD_600_ = 0.3 and incubated for an additional 1 h at 37°C with shaking at 100 rpm under microaerophilic conditions (luminescence-based GSH assay) or centrifuged (18,000 x *g*, 5 min, room temperature) and resuspended in fresh medium at OD_600_ = 1.0 (LC-MS analyses) prior to infection. Bacterial cultures, or an equivalent volume of 10% (v/v) FBS-HI in Brucella broth (mock infection control), were diluted in co-culture medium (DMEM or RPMI 1640 supplemented with 10% (v/v) Brucella broth and 5% (v/v) FBS-HI) to achieve an MOI of 35 or 50. The gastric cell culture medium was replaced with the appropriate co-culture medium, and the infected cells were incubated at 37°C in a humidified 5% CO_2_ incubator for 3–20 h as specified. To verify the MOI and assess bacterial growth during infection, a portion of the *H*. *pylori*-containing co-culture medium was collected at the start and end of each experiment and serially diluted in Brucella broth supplemented with 10% (v/v) FBS-HI for the enumeration of bacterial CFU. Gastric cell viability was quantified by Trypan Blue (Thermo Fisher Scientific) exclusion using a Countess II FL automated cell counter (Thermo Fisher Scientific).

### Antioxidant treatment

AGS cells were pretreated with either 100 μM or 500 μM 2-mercaptoethanol (Sigma) or 5 mM N-acetylcysteine (Sigma) in DMEM containing 10% (v/v) FBS-HI for 1 h prior to infection. At the time of infection, the cell culture medium was replaced with co-culture medium containing an equivalent concentration of either 2-mercaptoethanol or N-acetylcysteine.

### Measurement of intracellular GSH via luminescence-based assay

Gastric cells were washed once with Dulbecco’s PBS (DPBS; HyClone) and collected via dissociation with TrypLE Express (Gibco). Cells were pelleted by centrifugation (300 x *g*, 3 min, room temperature in 15-ml conical tubes (Corning), followed by 21,000 x *g*, 2 min, room temperature in microcentrifuge tubes). Harvested cells were resuspended in PBS (Gibco), transferred to white-walled 96-well plates (Corning), and analyzed using the GSH-Glo Assay kit (Promega) according to the manufacturer’s instructions. Intracellular GSSG and total GSH concentrations were measured using the GSH/GSSG-Glo Assay kit (Promega) according to manufacturer’s instructions. Luminescence was measured using a SpectraMax i3x Multi-Mode Microplate Reader.

### *H*. *pylori* growth curves

Overnight cultures of WT, Δ*gGT*, and Δ*gGT∷gGT H*. *pylori* G27 were grown for 16 h at 37°C and diluted to OD_600_ = 0.1 in fresh Brucella broth containing 10% (v/v) FBS HI with or without 50 μg/mL light GSH (Sigma). Subcultures were grown for an additional 22 h at 37°C. CFU were enumerated directly after subculturing (t = 0) and again following 4, 8, and 22 h of growth.

### Metabolic labeling of *H*. *pylori* with [^13^C_2_, ^15^N_1_]-GSH

Overnight cultures of *H*. *pylori* were grown for 16 h at 37°C and diluted to OD_600_ = 0.1 in Brucella broth containing 10% (v/v) FBS HI and 50 μg/mL glutathione (glycine-^13^C_2_, ^15^N) (95%+ purity, Cambridge Isotope Laboratories; aka [^13^C_2_, ^15^N_1_]-GSH). Subcultures were grown for an additional 8 h at 37°C, and a portion of each culture was collected for the enumeration of CFU. Cultures were then normalized by OD_600_ and pelleted by centrifugation (18,000 x *g*, 10 min, room temperature). Cell pellets were washed twice with PBS (Fisher Scientific) and stored at -80°C prior to further analysis.

### Metabolic labeling of gastric cells with [2-^13^C_1_]-Gly

AGS cells were treated with 0–50 μg/mL 2-^13^C glycine (99% purity, Cambridge Isotope Laboratories; aka [2-^13^C_1_]-Gly) in DMEM supplemented with 10% (v/v) FBS HI for 24 h. To assess biosynthesis of [^13^C_1_]-GSH, AGS cells were washed twice in DPBS (Hyclone), and collected via dissociation with TrypLE Express. Cells were pelleted by centrifugation (300 x *g*, 3 min, room temperature in 15-ml conical tubes (Corning), followed by 21,000 x *g*, 2 min, room temperature in microcentrifuge tubes) and stored at -80°C until further analysis. To assess [^13^C_1_]-GSH or [^13^C_1_]-Cys-Gly levels in cells co-cultured with *H*. *pylori*, AGS cells pretreated with 0 or 50 μg/mL [2-^13^C_1_]-Gly were subsequently washed twice with DPBS and infected at an MOI of 35 for 16 h. Following infection, conditioned cell culture medium containing non-adherent *H*. *pylori* was collected and pelleted by centrifugation (18,000 x *g*, 10 min, room temperature). Bacterial cell pellets were stored at -80°C until further analysis. AGS cells were treated with 400 μg/mL kanamycin (Thermo Fisher Scientific) in DMEM for 1 h and washed once with DPBS. Cells were collected via dissociation by TrypLE Express and pelleted by centrifugation (300 x *g*, 3 min, room temperature in 15-ml conical tubes, followed by 21,000 x *g*, 2 min, room temperature in microcentrifuge tubes) and stored at -80°C until further analysis.

### Metabolite extraction and thiol labeling

Frozen *H*. *pylori* or AGS cell pellets were thawed on ice and resuspended in 500 μL ice-cold thiol extraction buffer (50% (v/v) acetonitrile (ACN; Millipore), 50 mM HEPES pH 8 (Sigma), 1 mM TCEP (Sigma), and 1 mM diethylenetriaminepentaacetic acid (DTPA; Sigma)) for 30 min at -20°C. Samples were centrifuged (21,000 x *g*, 10 min, 4°C), and the supernatants were subsequently transferred to glass vials (Fisher Scientific). L-cysteine (1-^13^C) (99% purity, Cambridge Isotope Laboratories; 1 μg) was added to each sample and used as an internal standard to confirm similar processing across all replicates. Samples were treated with 35 μL of a 74 mM solution of monobromobimane (mBBr; Santa Cruz Biotechnology) in 100% ACN for 15 min at 60°C, protected from light, then frozen and lyophilized overnight at -80°C. Samples were reconstituted in 100% methanol (Avantor) and centrifuged (21,000 x *g*, 10 min, 4°C). Supernatants were analyzed immediately thereafter by LC-MS or LC-MS/MS.

### LC-MS and LC-MS/MS analysis

LC-MS and LC-MS/MS analysis was performed using an Agilent 6546 liquid chromatography/ quadrupole time-of-flight (LC/Q-TOF) mass spectrometer with an Agilent Jet Stream electrospray ionization (ESI) source coupled to an Agilent 1290 Infinity II ultra-high performance liquid chromatography (UHPLC) system. Samples were separated using a Kinetex C18 (100 Å) 5 μm (250 x 4.6 mm) column (Phenomenex) under the following conditions: flow rate 0.7 mL/min; mobile phase, water/ACN gradient containing 0.1% (v/v) formic acid: 30 min 0–100% (v/v) ACN, 5 min at 100% (v/v) ACN, 1 min at 100–5% (v/v) ACN, 2 min at 5% (v/v) ACN, 2.5 min post-time at 5% (v/v) ACN. The Q-TOF instrument was run in positive scanning mode (50–1700 *m/z*) using the following source parameters: capillary voltage, 4000 V; nozzle voltage, 2000 V; gas temperature, 325°C; gas flow, 5 L/min; nebulizer, 20 psi; sheath gas temperature, 275°C; sheath gas flow, 12 L/min. Mass calibration was achieved using a second ionization source and constant flow (1.5 ml min^-1^) of reference solution (121.0509 *m/z* and 922.0098 *m/z*). Tandem MS/MS analyses of bimane-labeled light GSH (mB-GSH) and heavy GSH (mB-[^13^C_1_]-GSH) were performed using identical HPLC conditions and Q-TOF source parameters as described above. Targeted MS/MS of *m/z* 498.1653 or *m/z* 499.1687 was performed using a mass tolerance of 100 ppm and a collision energy of 10, 20, or 30 eV. All data were analyzed using Agilent MassHunter Quantitative Analysis software version 10.0. The natural isotopic *m/z* distribution of bimane-labeled GSH (mB-GSH) was predicted using Perkin Elmer ChemDraw Professional software version 21.0.0.28.

### Statistical analyses

The numerical data and statistical analyses shown in figure panels 1A, 1B, 1C, 1D, 2A, 2B, 3A, 3B, 3C, 3D, 3E, 4B, 4C, 4D, 4E, 4F, S1A, S1B, S2, S3, S4, S5, S7A, S7B, S7C, S7E, S8A, S8B, S9A, and S9B can be found in S1 Data. GraphPad Prism (v.9) was used to generate graphs and perform statistical analyses. Differences between two groups of data were analyzed using a two-tailed, unpaired *t* test. Differences between multiple groups of data were assessed by analysis of variance (ANOVA) followed by Šídák’s multiple comparisons test (comparisons across specified datasets) or Dunnett’s multiple comparisons test (comparisons to a control dataset). Differences with *P* < 0.05 were regarded as statistically significant.

## Supporting information

S1 Data Excel spreadsheet containing, in separate sheets, the numerical data and statistical analyses for figure panels 1A, 1B, 1C, 1D, 2A, 2B, 3A, 3B, 3C, 3D, 3E, 4B, 4C, 4D, 4E, 4F, S1A, S1B, S2, S3, S4, S5, S7A, S7B, S7C, S7E, S8A, S8B, S9A, and S9B. (XLSX)

S1 Fig Antioxidant treatment does not inhibit *H*. *pylori-*induced depletion of reduced GSH from AGS cells. (A) Levels of total GSH in *H*. *pylori*-infected (*H*. *pylori* G27, MOI 50, 10 h) and uninfected AGS cells pre-incubated with BME or medium alone (untreated) for 1 h (left), normalized by the total number of live AGS cells in each condition (right). AGS cells were also incubated with BME or medium alone for the duration of infection. (B) CFU of *H*. *pylori* in conditioned culture media from (A) and Fig 2B. Data represent three independent experiments. Each circle represents an independent experiment. Error bars represent means ± SD. *P < 0.05; **P < 0.01; ****P < 0.0001; ns, not significant. A two-way ANOVA with Šídák’s multiple comparisons test was used for (A), and a one-way ANOVA with Dunnett’s multiple comparisons test was used for (B).
(TIF)

S2 Fig *gGT* is not expressed by *H*. *pylori* Δ*gGT*. RT-qPCR analysis of *gGT* expression normalized to *ppk* expression in *H*. *pylori* G27 Δ*gGT* and Δ*gGT∷gGT*. *gGT* mRNA levels are reported relative to *gGT* expression in WT *H*. *pylori* G27. Data represent three independent experiments. Each circle represents an independent experiment. Error bars represent means ± SD. *P < 0.05 by two-tailed t-test.
(TIF)

S3 Fig Deletion of *H*. *pylori gGT*, *cagA*, or *vacA* does not alter bacterial viability during *H*. *pylori* infection of AGS cells. CFU of WT, Δ*gGT*, Δ*gGT∷gGT*, Δ*cagA*, and Δ*vacA H*. *pylori* G27 in conditioned culture media from *H*. *pylori-*infected AGS cells (MOI 35, 16 h). Data represent three independent experiments. Each circle represents an independent experiment. Error bars represent means ± SD. ns, not significant. A one-way ANOVA with Dunnett’s multiple comparisons test was used.
(TIF)

S4 Fig *H*. *pylori* growth is not altered in medium supplemented with light GSH. WT, Δ*gGT*, and Δ*gGT∷gGT H*. *pylori* G27 were grown in medium supplemented with light GSH and CFU were enumerated at the indicated time points. Each circle represents a technical replicate from a single experiment. Growth curve analyses were performed three separate times with consistent results. ns, not significant by two-way ANOVA with Tukey’s multiple comparisons test.
(TIF)

S5 Fig Deletion of *H*. *pylori gGT* does not alter bacterial growth in medium supplemented with [^13^C_2_, ^15^N_1_]-GSH. WT, Δ*gGT*, or Δ*gGT∷gGT H*. *pylori* G27 were grown in medium supplemented with heavy GSH ([^13^C_2_, ^15^N_1_]-GSH) for 8 h prior to the enumeration of CFU. Data represent three independent experiments. Each circle represents an independent experiment. Error bars represent means ± SD. ns, not significant. A one-way ANOVA with Dunnett’s multiple comparisons test was used.
(TIF)

S6 Fig The γ-glutamyl cycle in eukaryotic cells. GSH biosynthesis begins with the production of γ-glutamylcysteine from glutamate and cysteine in a process catalyzed by γ-glutamylcysteine synthase (GCLC) [69]. A second enzyme, GSH synthetase (GSS), adds glycine to the γ-glutamylcysteine dipeptide to produce GSH. Cation transport regulator 1 (CHAC1), a γ-glutamyl cyclotransferase, hydrolyzes GSH to 5-oxoproline and Cys-Gly. 5-oxoproline is cleaved by 5-oxoprolinase (OPLAH) to yield glutamate, whereas Cys-Gly is cleaved by specific peptidases to yield cysteine and glycine.
(TIF)

S7 Fig AGS cells treated with [2-^13^C_1_]-Gly synthesize mB-[^13^C_1_]-GSH. (A) AGS cells were incubated with [2-^13^C_1_]-Gly at the indicated concentration or with medium alone (untreated) for 24 h. EIC spectra (*m/z* 499.1687, corresponding to mB-[^13^C_1_]-GSH) of AGS cell extracts treated with mBBr. (B) Ion counts of mB-[^13^C_1_]-GSH for (A), normalized by the total number of live cells per condition. (C) MS^2^ fragmentation spectra of an unlabeled GSH standard treated with mBBr (mB-GSH, *m/z* 498.1653; left) or of extracts from AGS cells incubated with 50 μg/mL [2-^13^C_1_]-Gly for 24 h prior to mBBr labeling (mB-[^13^C_1_]-GSH, *m/z* 499.1687; right). (D) Predicted natural isotopic *m/z* distribution of mB-GSH. Predicted *m/z* value corresponding to that detected in (E) (left) is highlighted in red. (E) Mass spectra (retention time 7.9 min) of an unlabeled GSH standard treated with mBBr (mB-GSH; left) or of extracts from AGS cells incubated with 50 μg/mL [2-^13^C_1_]-Gly for 24 h prior to mBBr labeling (mB-[^13^C_1_]-GSH; right). Data in (A) and (B) represent three technical replicates from a single experiment, and each circle in (B) represents a single replicate. Data in (C) and (E) are representative of a single experiment that was repeated twice with similar results. Error bars represent means ± SD. *P < 0.05; ns, not significant. Multiple unpaired t-tests were used for (B).
(TIF)

S8 Fig Pretreatment of AGS cells with [2-^13^C_1_]-Gly does not alter AGS or bacterial viability following infection with *H*. *pylori*. (A) AGS cells were incubated with 50 μg/mL [2-^13^C_1_]-Gly for 24 h and then infected with WT, Δ*gGT*, or Δ*gGT∷gGT H*. *pylori* (G27, MOI 35, 16 h) prior to quantification of AGS cell viability. (B) CFU of WT, Δ*gGT*, and Δ*gGT∷gGT H*. *pylori* G27 in conditioned culture media from (A). Data represent three independent experiments. Each circle represents an independent experiment. Error bars represent means ± SD. ns, not significant. A one-way ANOVA with Dunnett’s multiple comparisons test was used for (A) and (B).
(TIF)

S9 Fig mB-[^13^C_1_]-Cys-Gly is not detected in *H*. *pylori* harvested from conditioned co-culture media. (A) AGS cells treated with [2-^13^C_1_]-Gly for 24 h and untreated controls were infected with WT *H*. *pylori* G27 (MOI 35) for 16 h. EIC spectra (*m/z* 370.1261, corresponding to mB-[^13^C_1_]-Cys-Gly) of *H*. *pylori* cell extracts treated with mBBr. (B) Ion counts of mB-[^13^C_1_]-Cys-Gly for (A), normalized by CFU. Data represent two independent experiments. Each circle in (B) represent*s* an independent experiment. Error bars represent means ± SD. ns, not significant. A two-tailed unpaired t-test was used for (B).
(TIF)

S1 Table Strain list. (DOCX)

S2 Table Primer list. (DOCX)

S3 Table Plasmid list. (DOCX)
