# Supplementary figures and images for: Isotope tracing reveals bacterial catabolism of host-derived glutathione during Helicobacter pylori infection
Source: PLoS Pathog. 2023 Jul 26;19(7):e1011526. doi: 10.1371/journal.ppat.1011526 (PMC10406306; doi:10.1371/journal.ppat.1011526)

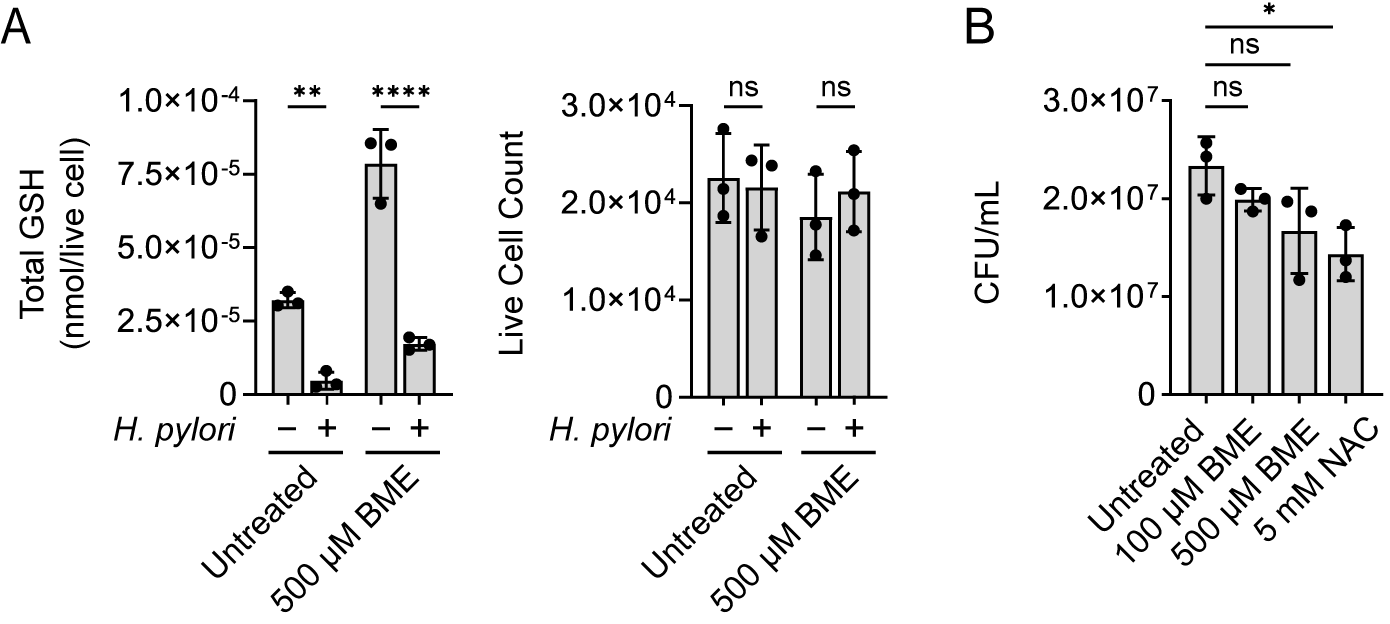

Supplement: S1 Fig — (A) Levels of total GSH in H. pylori-infected (H. pylori G27, MOI 50, 10 h) and uninfected AGS cells pre-incubated with BME or medium alone (untreated) for 1 h (left), normalized by the total number of live AGS cells in each condition (right). AGS cells were also incubated with BME or medium alone for the duration of infection. (B) CFU of H. pylori in conditioned culture media from (A) and Fig 2B. Data represent three independent experiments. Each circle represents an independent experiment. Error bars represent means ± SD. *P < 0.05; **P < 0.01; ****P < 0.0001; ns, not significant. A two-way ANOVA with Šídák’s multiple comparisons test was used for (A), and a one-way ANOVA with Dunnett’s multiple comparisons test was used for (B). (TIF) [file ppat.1011526.s002.tif]

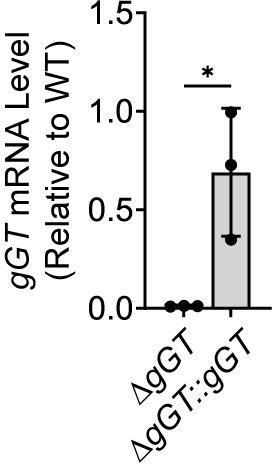

Supplement: S2 Fig — RT-qPCR analysis of gGT expression normalized to ppk expression in H. pylori G27 ΔgGT and ΔgGT∷gGT. gGT mRNA levels are reported relative to gGT expression in WT H. pylori G27. Data represent three independent experiments. Each circle represents an independent experiment. Error bars represent means ± SD. *P < 0.05 by two-tailed t-test. (TIF) [file ppat.1011526.s003.tif]

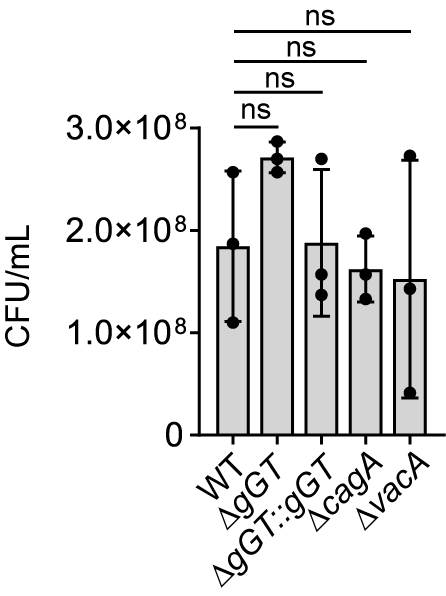

Supplement: S3 Fig — CFU of WT, ΔgGT, ΔgGT∷gGT, ΔcagA, and ΔvacA H. pylori G27 in conditioned culture media from H. pylori-infected AGS cells (MOI 35, 16 h). Data represent three independent experiments. Each circle represents an independent experiment. Error bars represent means ± SD. ns, not significant. A one-way ANOVA with Dunnett’s multiple comparisons test was used. (TIF) [file ppat.1011526.s004.tif]

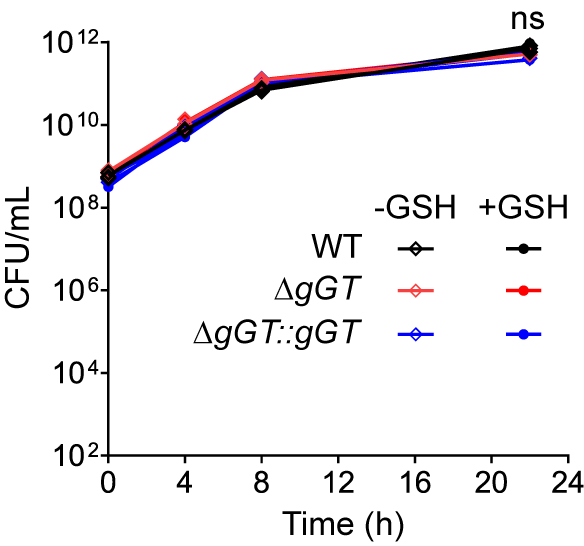

Supplement: S4 Fig — WT, ΔgGT, and ΔgGT∷gGT H. pylori G27 were grown in medium supplemented with light GSH and CFU were enumerated at the indicated time points. Each circle represents a technical replicate from a single experiment. Growth curve analyses were performed three separate times with consistent results. ns, not significant by two-way ANOVA with Tukey’s multiple comparisons test. (TIF) [file ppat.1011526.s005.tif]

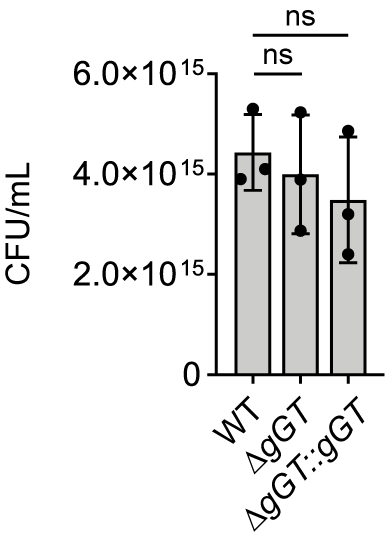

Supplement: S5 Fig — WT, ΔgGT, or ΔgGT∷gGT H. pylori G27 were grown in medium supplemented with heavy GSH ([13C2, 15N1]-GSH) for 8 h prior to the enumeration of CFU. Data represent three independent experiments. Each circle represents an independent experiment. Error bars represent means ± SD. ns, not significant. A one-way ANOVA with Dunnett’s multiple comparisons test was used. (TIF) [file ppat.1011526.s006.tif]

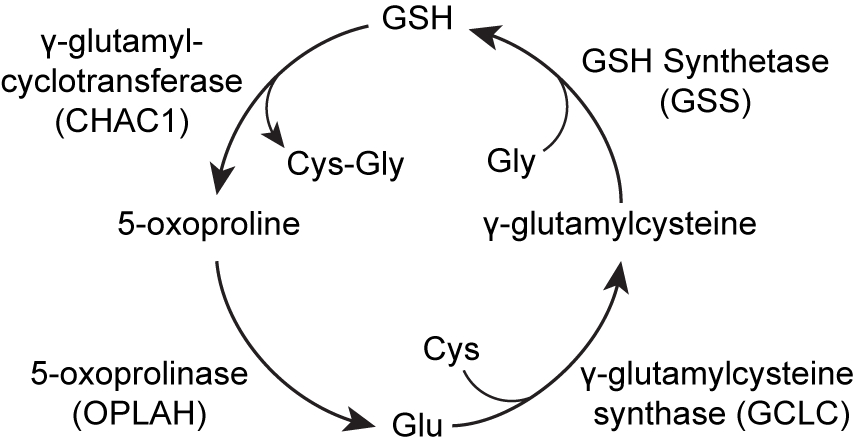

Supplement: S6 Fig — GSH biosynthesis begins with the production of γ-glutamylcysteine from glutamate and cysteine in a process catalyzed by γ-glutamylcysteine synthase (GCLC) [69]. A second enzyme, GSH synthetase (GSS), adds glycine to the γ-glutamylcysteine dipeptide to produce GSH. Cation transport regulator 1 (CHAC1), a γ-glutamyl cyclotransferase, hydrolyzes GSH to 5-oxoproline and Cys-Gly. 5-oxoproline is cleaved by 5-oxoprolinase (OPLAH) to yield glutamate, whereas Cys-Gly is cleaved by specific peptidases to yield cysteine and glycine. (TIF) [file ppat.1011526.s007.tif]

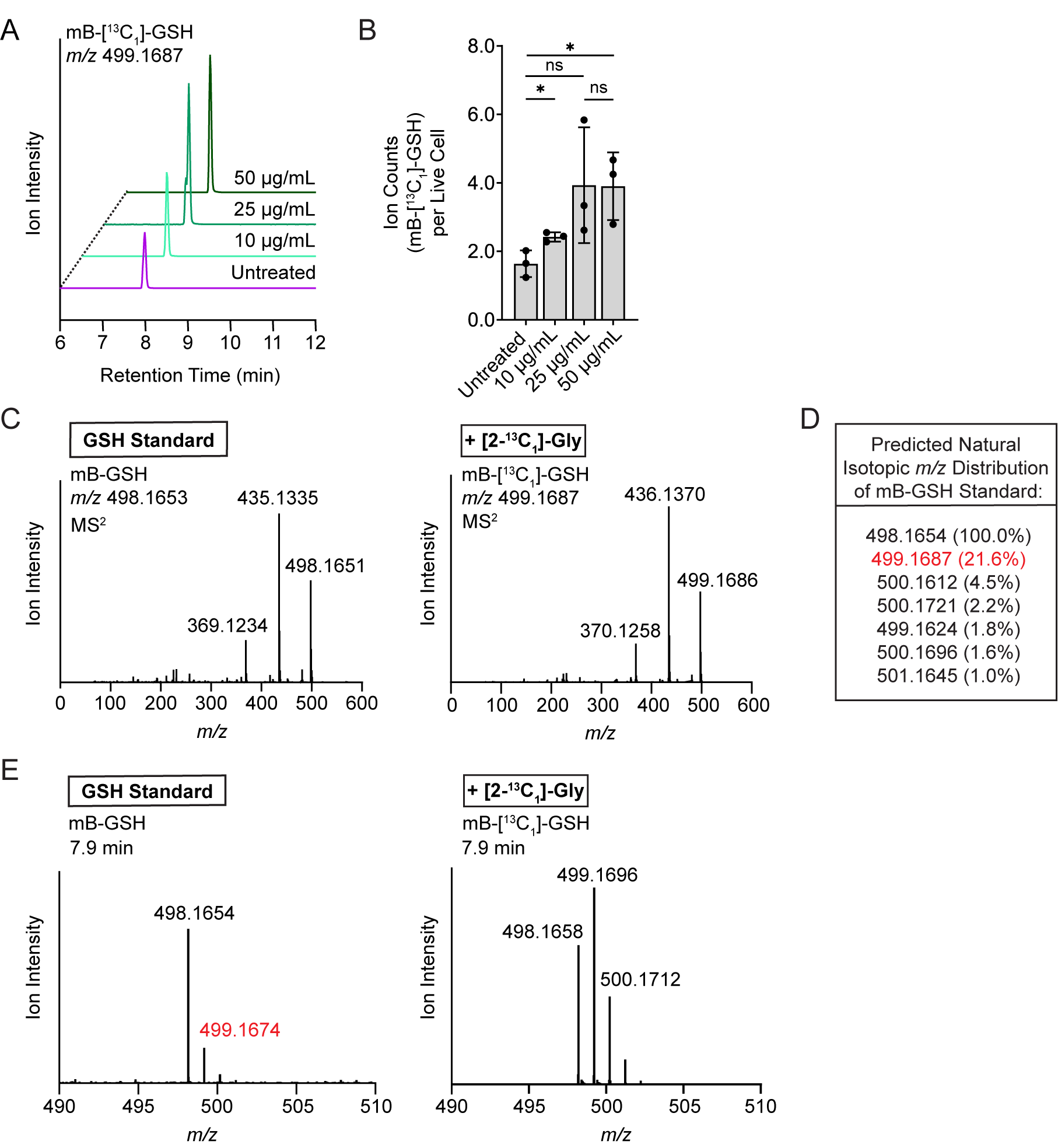

Supplement: S7 Fig — (A) AGS cells were incubated with [2-13C1]-Gly at the indicated concentration or with medium alone (untreated) for 24 h. EIC spectra (m/z 499.1687, corresponding to mB-[13C1]-GSH) of AGS cell extracts treated with mBBr. (B) Ion counts of mB-[13C1]-GSH for (A), normalized by the total number of live cells per condition. (C) MS2 fragmentation spectra of an unlabeled GSH standard treated with mBBr (mB-GSH, m/z 498.1653; left) or of extracts from AGS cells incubated with 50 μg/mL [2-13C1]-Gly for 24 h prior to mBBr labeling (mB-[13C1]-GSH, m/z 499.1687; right). (D) Predicted natural isotopic m/z distribution of mB-GSH. Predicted m/z value corresponding to that detected in (E) (left) is highlighted in red. (E) Mass spectra (retention time 7.9 min) of an unlabeled GSH standard treated with mBBr (mB-GSH; left) or of extracts from AGS cells incubated with 50 μg/mL [2-13C1]-Gly for 24 h prior to mBBr labeling (mB-[13C1]-GSH; right). Data in (A) and (B) represent three technical replicates from a single experiment, and each circle in (B) represents a single replicate. Data in (C) and (E) are representative of a single experiment that was repeated twice with similar results. Error bars represent means ± SD. *P < 0.05; ns, not significant. Multiple unpaired t-tests were used for (B). (TIF) [file ppat.1011526.s008.tif]

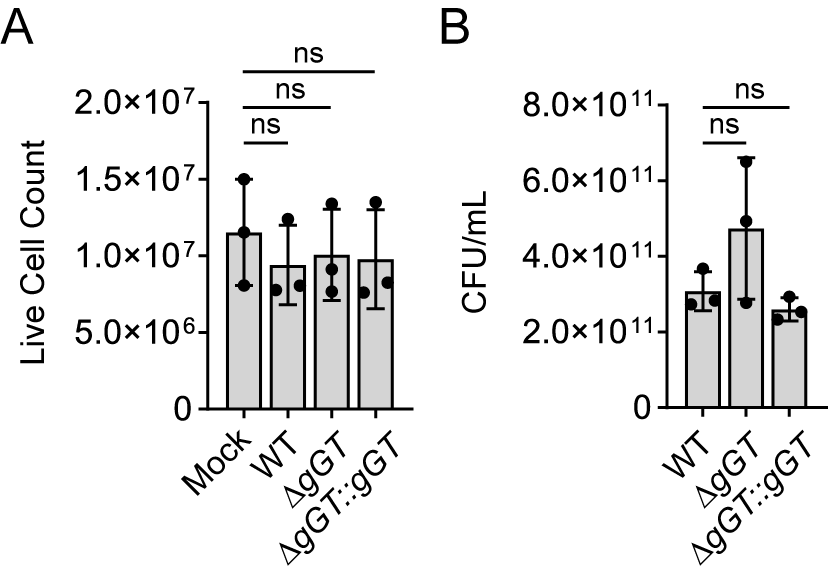

Supplement: S8 Fig — (A) AGS cells were incubated with 50 μg/mL [2-13C1]-Gly for 24 h and then infected with WT, ΔgGT, or ΔgGT∷gGT H. pylori (G27, MOI 35, 16 h) prior to quantification of AGS cell viability. (B) CFU of WT, ΔgGT, and ΔgGT∷gGT H. pylori G27 in conditioned culture media from (A). Data represent three independent experiments. Each circle represents an independent experiment. Error bars represent means ± SD. ns, not significant. A one-way ANOVA with Dunnett’s multiple comparisons test was used for (A) and (B). (TIF) [file ppat.1011526.s009.tif]

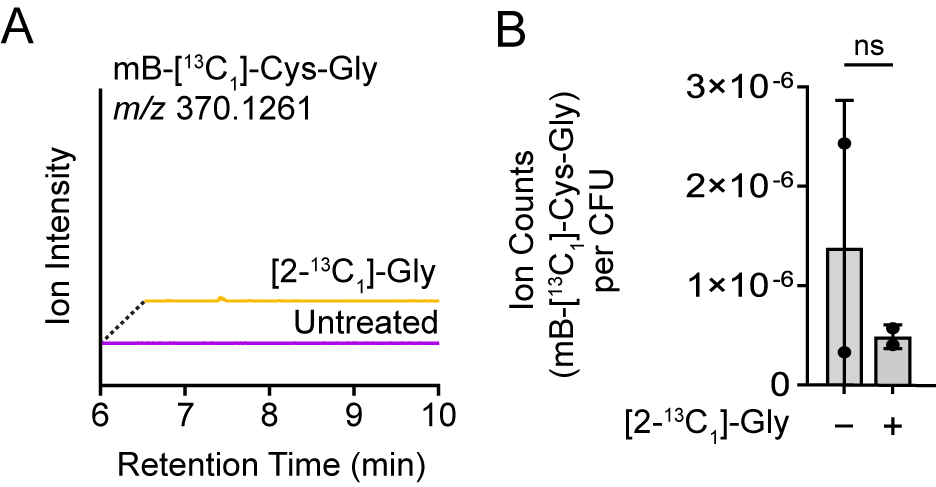

Supplement: S9 Fig — (A) AGS cells treated with [2-13C1]-Gly for 24 h and untreated controls were infected with WT H. pylori G27 (MOI 35) for 16 h. EIC spectra (m/z 370.1261, corresponding to mB-[13C1]-Cys-Gly) of H. pylori cell extracts treated with mBBr. (B) Ion counts of mB-[13C1]-Cys-Gly for (A), normalized by CFU. Data represent two independent experiments. Each circle in (B) represents an independent experiment. Error bars represent means ± SD. ns, not significant. A two-tailed unpaired t-test was used for (B). (TIF) [file ppat.1011526.s010.tif]
