# Supplementary material for: Isotope tracing reveals bacterial catabolism of host-derived glutathione during Helicobacter pylori infection
Source: PLoS Pathog. 2023 Jul 26;19(7):e1011526. doi: 10.1371/journal.ppat.1011526 (PMC10406306; doi:10.1371/journal.ppat.1011526)
Supplement: S1 Table — (DOCX) [file ppat.1011526.s011.docx]

**S1 Table. Strain list.**

| Strain | Genotype/Description | Reference/Source |
| --- | --- | --- |
| *Helicobacter pylori* G27 | WT strain of *H. pylori* isolated from a patient with peptic ulcer disease | [1] |
| *H. pylori* G27 ∆*gGT* | Marked deletion of *gGT* in strain G27 (KanR) | [1] |
| *H. pylori* G27 ∆*vacA* | Marked deletion of *vacA* in strain G27 (CmR) | This study |
| *H. pylori* G27 ∆*cagA* | Marked deletion of *cagA* in strain G27 (CmR) | This study |
| *H. pylori* G27 ∆*gGT∷gGT* | G27 ∆*gGT* strain complemented with *gGT* and chloramphenicol cassette at the native locus (CmR) | This study |
| *H. pylori* PMSS1 | WT strain of *H. pylori* isolated from a patient with duodenal ulcers | [2] |
| *H. pylori* PMSS1 ∆gGT | Marked deletion of *gGT* in strain PMSS1 (KanR) | [3] |
| *H. pylori* G27MA ∆*cagA* | Marked deletion of *cagA* in strain G27MA (CmR) | Gift from Manuel Amieva (Stanford University School of Medicine) [4] |

**References**

1. Schmees C, Prinz C, Treptau T, Rad R, Hengst L, Voland P, et al. Inhibition of T-cell proliferation by *Helicobacter pylori* gamma-glutamyl transpeptidase. Gastroenterology. 2007;132(5):1820-33.

2. Arnold IC, Lee JY, Amieva MR, Roers A, Flavell RA, Sparwasser T, et al. Tolerance rather than immunity protects from *Helicobacter pylori*-induced gastric preneoplasia. Gastroenterology. 2011;140(1):199-209.

3. Oertli M, Noben M, Engler DB, Semper RP, Reuter S, Maxeiner J, et al. *Helicobacter pylori* gamma-glutamyl transpeptidase and vacuolating cytotoxin promote gastric persistence and immune tolerance. Proc Natl Acad Sci USA. 2013;110(8):3047-52.

4. Tan S, Tompkins LS, Amieva MR. *Helicobacter pylori* usurps cell polarity to turn the cell surface into a replicative niche. PLoS Pathog. 2009;5(5):e1000407.
