# Supplementary material for: Isotope tracing reveals bacterial catabolism of host-derived glutathione during Helicobacter pylori infection
Source: PLoS Pathog. 2023 Jul 26;19(7):e1011526. doi: 10.1371/journal.ppat.1011526 (PMC10406306; doi:10.1371/journal.ppat.1011526)
Supplement: S2 Table — (DOCX) [file ppat.1011526.s012.docx]

**S2 Table. Primer list.**

| **Primer** | **Sequence (5' to 3')** | **Description** |
| --- | --- | --- |
| MB-14 | GTGCATGCAAGATGATTTAAACGTT | Forward primer for PCR amplification of the 604-bp upstream flanking region of *H. pylori* G27 *vacA* |
| MB-15 | ATCCACTTTTCAATCTATATCTTTCTTCCTTTCTTTTTGTAAAACGATT | Reverse primer for PCR amplification of the 604-bp upstream flanking region of *H. pylori* G27 *vacA* |
| MB-16 | CCCAGTTTGTCGCACTGATAAGGTATAGTTTCTAAATACCGCTCTTA | Forward primer for PCR amplification of the 576-bp downstream flanking region of *H. pylori* G27 *vacA* |
| MB-17 | GCGTTCAATTTCAGCGTGCTAGATT | Reverse primer for PCR amplification of the 576-bp downstream flanking region of *H. pylori* G27 *vacA* |
| MB-71 | GCAACTCCATAGACCACTAAAGAAACTTTTTTTG | Forward primer for PCR amplification of the 502-bp upstream flanking region of *H. pylori* G27 *cagA* |
| MB-74 | CCTAGAAATAGCTACATTCAAGCGTTTAGAAT | Reverse primer for PCR amplification of the 500-bp downstream flanking region of *H. pylori* G27 *cagA* |
| MB-1 | GATATAGATTGAAAAGTGGAT | Forward primer for PCR amplification of the chloramphenicol-resistance cassette *cat* from G27MA ∆*cagA* |
| MB-2 | TTATCAGTGCGACAAACTGGG | Reverse primer for PCR amplification of the chloramphenicol-resistance cassette *cat* from G27MA ∆*cagA* |
| MB-39 | GCAATTGCAATACGACAGCGTGGAT | Forward primer for PCR amplification of the 501-bp upstream flanking region of *H. pylori* G27 *gGT* |
| MB-42 | GGTACACCCCTACCATTTTCATACATGATCCC | Reverse primer for PCR amplification of the 503-bp downstream flanking region of *H. pylori* G27 *gGT* |
| MJB-21 | AGCGAGTTATCCGCCCATTA | Forward primer for qRT-PCR amplification of *gGT* in *H. pylori* G27 |
| MJB-22 | TGGACGACTGCTAGAGCAAA | Reverse primer for qRT-PCR amplification of gGT in H. pylori G27 |
| ABS-75 | CGCGGATTTAATGCCCAGAA | Forward primer for qRT-PCR amplification of *ppk* in *H. pylori* G27 |
| ABS-76 | CTATCCACTAGGGCGTTGGC | Reverse primer for qRT-PCR amplification of *ppk* in *H. pylori* G27 |
