# Supplementary material for: Isotope tracing reveals bacterial catabolism of host-derived glutathione during Helicobacter pylori infection
Source: PLoS Pathog. 2023 Jul 26;19(7):e1011526. doi: 10.1371/journal.ppat.1011526 (PMC10406306; doi:10.1371/journal.ppat.1011526)
Supplement: S3 Table — (DOCX) [file ppat.1011526.s013.docx]

**S3 Table. Plasmid list.**

| **Plasmid** | **Description** | **Reference/Source** |
| --- | --- | --- |
| pUC57_*gGT::gGT-cat* | pUC57 vector containing the *gGT* revertant complement construct at the EcoRV restriction site | This study |
